# Do children’s previous dental experience and fear affect their perceived oral health-related quality of life (OHRQoL)?

**DOI:** 10.1186/s12903-017-0338-9

**Published:** 2017-01-16

**Authors:** Leena Merdad, Azza A. El-Housseiny

**Affiliations:** 1 Department of Dental Public Health, Faculty of Dentistry, King Abdulaziz University, P.O. Box 80209, 21589 Jeddah, Saudi Arabia; 2 Department of Pediatric Dentistry, Faculty of Dentistry, King Abdulaziz University, Jeddah, Saudi Arabia; 3 Department of Pediatric Dentistry, Faculty of Dentistry, Alexandria University, Alexandria, Egypt

**Keywords:** Oral health-related quality of life, CPQ, CPQ_11–14_, Children, Dental fear, Dental experience, predictors

## Abstract

**Background:**

Oral health-related quality of life (OHRQoL) has been used to describe the consequences of oral health conditions and treatments in children. A better understanding of OHRQoL and its relationship with dental fear and previous dental experience is necessary to improve children’s oral health status. The aim of this study was to investigate the association of dental history and experience with dental fear and the OHRQoL of children aged 11 to 14 years.

**Methods:**

A cross-sectional study was conducted using a multi-stage stratified sample of 1,312 middle school children. Information regarding OHRQoL was collected from the children using the Child Perceptions Questionnaire (CPQ_11–14_), and information regarding dental fear was collected using the Children’s Fear Survey Schedule-Dental Subscale (CFSS-DS). Information on past dental experiences and sociodemographic data were collected from the parents using self-administered questionnaires. Dental examinations were performed to assess caries experience.

**Results:**

The multivariable model indicated that dental fear was the strongest predictor of OHRQoL as the fearful children had on average CPQ_11–14_ scores that were 10 units higher than those of the non-fearful children. Regarding past dental experience, pain as the reason for the most recent dental visit was associated with poor OHRQoL, while receiving a filling during the previous dental visits was significantly associated with better OHRQoL. In addition, a larger number of siblings, a lower family income, a lower paternal education level, health problems and prior hospitalization were significantly associated with poor OHRQoL.

**Conclusion:**

This study identified that dental fear and some factors related to previous dental experience are associated with OHRQoL. In dental practice, children with dental fear should be identified, guided and treated early to avoid deterioration of their OHRQoL.

**Electronic supplementary material:**

The online version of this article (doi:10.1186/s12903-017-0338-9) contains supplementary material, which is available to authorized users.

## Background

It is important to assess the influence of oral health on the everyday life of children because oral diseases may not only limit children’s current physical, social and psychological well-being but may also affect their future development and academic achievement. Oral health-related quality of life (OHRQoL) is a multidimensional construct that consists of subjective assessments of oral health, emotional and functional well-being and self-esteem [1]. It has commonly been used to describe the outcomes of oral health conditions and treatments in children [1]. The late childhood/pre-adolescence stage is frequently characterized by a potentially high caries rate, a tendency toward poor nutritional habits, dental phobia, eating disorders, pre-occupation with others’ views and unique social and psychological needs [2]. A better understanding of OHRQoL and its influencing dental and clinical factors in pre-adolescent children is necessary to provide them with optimum oral health care and treatment and improve their oral health.

Many questionnaires have been developed to assess OHRQoL in children. The Child Perceptions Questionnaire (CPQ) is one of the most commonly used self-report scales [3]. This questionnaire was developed for and validated in children in Canada and showed adequate reliability and validity [4]. The CPQ items cover four health domains: oral symptoms, functional limitations, emotional well-being and social well-being [4]. The CPQ has been translated into many languages and has been adapted and validated for other cultures [3].

Dental fear and previous dental experience play a significant role in dental practice. Dental fear and anxiety are not uncommon among children and adolescents and present a challenge for dentists [5, 6]. Children’s dental anxiety has been recognized as a factor associated with behavior management problems [6] and as the most important factor in whether children will require general anesthesia during dental treatment [7]. Previous dental experience has also been reported to be related to children’s negative behavior at the dental clinic [8].

The relationship between previous dental experience and ORHQoL has been minimally investigated in the literature. A few studies have investigated the relationship between patterns of dental visits and OHRQoL [9, 10]. A study of Brazilian adults reported that compared with subjects who visit the dentist regularly, those who did not reported poor OHRQoL [9]. Studies on dental fear and OHRQoL are also limited, and the mechanism behind the relationship is unclear. A low to moderate correlation was found between dental anxiety/fear and OHRQoL in subjects aged 16 years and older [11]. A study that investigated dental fear in 11- to 14-year-olds in relation to OHRQoL suggested that dental fear negatively influenced children’s OHRQoL. In addition, the study found that the association between the social well-being and emotional well-being domains of the OHRQoL and dental fear was modified by orthodontic treatment [12]. Regarding caries experience, the relationship between caries and OHRQoL remains controversial. Some studies have reported a significant association between caries and poor OHRQoL [13], while others have reported no association [14].

Few studies have investigated the association between previous dental experience and the OHRQoL of pre-adolescent children and the possible impairment effect of dental fear. Thus, the main aim of this study was to investigate the factors associated with OHRQoL among children aged 11 to 14 years, with a focus on past dental history, experience and fear.

## Methods

### Study design & sampling

This cross-sectional analytical study was undertaken between September 2013 and May 2014 in the city of Jeddah, Saudi Arabia. Using a linear regression model and conservatively assuming a small effect size (0.02) with 18 predictors that have been reported in the literature to be associated with OHRQoL, a significance level of 0.05 and a power of 0.9, the estimated sample size was 1,273 children. The sample size was increased to 1,500 to account for non-responders.

A multistage stratified random sampling technique was used to select the population from children enrolled in middle schools in the city. According to the Ministry of Education, a total of 109,710 children (59,460 male and 50,250 female) were registered in 431 middle schools. The schools were randomly selected from the city’s school list, which was stratified according to gender (male or female), funding source (public or private) and district (North, East, South or West). A total of 16 middle schools were selected. In each school, one class from each grade (7th to 9th grades) was randomly selected for inclusion in the study. Students aged 11 to 14 years old in the selected classrooms were included in the study. The response rate was approximately 87%. Ethical approval was obtained from the Research Ethics Committee, Faculty of Dentistry, King Abdulaziz University. Permission to conduct the study was also obtained from the Ministry of Education and the schools’ principals.

### Questionnaires

Two questionnaires were used in this study. The first was the child questionnaire, which consisted of two parts. The first part was the Child Perceptions Questionnaire (CPQ_11–14_), which is used to measure OHRQoL in children aged 11 to 14 years [4]. It is composed of 37 items that cover four health domains: oral symptoms, functional limitations, emotional well-being and social well-being. In this study, the validated Arabic version of the CPQ_11–14_ questionnaire, which has been reported to have good psychometric properties [15], was used. The Arabic version is composed of 36 items; the question about difficulties playing musical instruments was omitted because the participating children rarely studied music [15]. The participants were asked about the frequency of certain events in the last three months. They had 5 response categories to choose from: 0 = Never, 1 = Once or twice, 2 = Sometimes, 3 = Often and 4 = Every day or almost every day. The total score ranges from a minimum of 0 to a maximum of 144, and higher scores indicate worse OHRQoL.

The second part of the child questionnaire was the Children’s Fear Survey Schedule-Dental Subscale (CFSS-DS), which is the most commonly, used scale to measure dental fear in children [16]. The CFSS-DS is composed of 15 items related to different types of dental treatment, such as “injections” and “drilling”. Each item is rated on a 5-point Likert scale with scores ranging from 1, “not afraid at all”, to 5, “very afraid”. The total score ranges from a minimum of 15 to a maximum of 75. A higher score reveals higher dental fear [17]. The validated Arabic version of CFSS-DS was used in this study [18].

The second questionnaire used in this study was the parent questionnaire (Additional file 1), which was used to assess the different factors associated with the child’s OHRQoL. It included questions about the child’s age, gender, birth order, number of siblings, family income, parents’ education, and general medical history (including the presence of medical health problems and any prior hospitalization). It also included questions about dental history and dental experiences (including the frequency and patterns of dental visits, the reason for the most recent dental visit and the procedures involved and behavior during previous visits).

A package was sent to the children’s parents that included a letter explaining the objectives of the study, the study questionnaires and the consent form. The investigators collected the parent questionnaires from the students, and only those with a signed informed consent were asked to complete the child questionnaire. Instructions on how to complete both the CPQ_11–14_ and CFSS-DS were given to the children by a trained investigator, who read each item aloud in the classroom, explained unclear questions and made sure that the children completed the items independently.

### Dental examination

After the children completed the questionnaires, dental examinations were performed to determine the presence of caries. The examination was conducted using a sterile, flat-surfaced mouth mirror and a Community Periodontal Index (CPI) probe (Nordent, Elk Grove Village, IL, USA) under adequate lighting and proper infection control measures. The children were seated on a chair with a backrest in front of the examiner. The index for decayed, missing and filled primary teeth (dmft) and permanent teeth (DMFT) was recorded according to the 1997 WHO criteria [19]. Caries examinations were performed out by two trained and calibrated examiners (Kappa = 0.93).

### Variables

The main outcome in this study was the total CPQ_11–14_ score. It was calculated for each child by summing the score for all the items of the CPQ_11–14_. The predictors measured in this study were the child’s age, gender, birth order, and number of siblings; family income; parents’ education; history of health problems; history of hospitalization; dental visits, dental visits in the last 3 months; main reasons for the most recent dental visit; main procedure during the previous dental visits; history of poor behavior reported by parents during the previous dental visits, dental fear, measured with the CFSS-DS; and caries in the primary and permanent dentition. Regarding the fear score, for each child, the total score was calculated by adding the fear scores for each of the 15 items on the CFSS-DS. A cutoff score of 32 was used to divide the children into fearful and non-fearful groups [20–22]. The D/d component of the DMFT/dmft was used to identify untreated carious teeth.

### Statistical analysis

Categorical data were described as frequencies and percentages, and continuous data were described as means and standard deviations (SD). The associations between the predictors and the total CPQ_11–14_ score were tested using *t*-tests and one-way analysis of variance (ANOVA). Tukey post-hoc tests were used if the one-way ANOVA showed significant differences between the groups. The association between the DMFT and CPQ_11–14_ scores was assessed using Pearson’s correlation. A stepwise linear regression with an entry of 0.05 and a removal of 0.1 was used to identify significant predictors of the total CPQ_11–14_ score. The significance level was set at 0.05. STATA version 13 (StataCorp, College Station, Texas, USA) was used to analyze the data.

## Results

### Descriptive statistics

A total of 1,312 children completed the questionnaire and participated in the study. The sociodemographic and health-related characteristics of the study population are displayed in Table 1. Approximately half of the children were 11 to 12 years old, and the other half were 13 to 14 years old. Females constituted 45% of the sample. The majority reported middle family income (71%). Regarding the educational level of the children’s mothers, a large percentage had a secondary education (39%); regarding the children’s fathers, 50% had a university education. Approximately 15% of children had health problems, with the majority of those children reporting a history of asthma (36%) or allergies (39%). Twenty-four percent reported a history of hospitalization, with the main causes reported as an operation (45%), followed by an emergency (40%).

**Table 1:** Characteristics of the study population

Sociodemographic variables	N	%	Dental history and experience	N	%
Age (years) (*n* = 1,312)			Dental visit ever (*n* = 1,295)		
11–12	653	50	Yes	1,158	89
13–14	659	50	No	137	11
Gender (*n* = 1,312)			Dental visit in the last 3 months (*n* = 1,271)		
Male	719	55	Yes	414	33
Female	593	45	No	857	67
Birth order (*n* = 1,312)			Reason for most recent dental visit (*n* = 1,287)^a^		
First	302	23	Regular check-up	101	8
2-3	510	39	Pain	782	61
4+	500	38	Filling	464	36
Number of siblings (*n* = 1,312)			Esthetic	148	12
0–3	455	35	Other	119	9
4–5	476	36	Procedure at previous dental visit/s (*n* = 1,286)^a^		
6+	381	29	Examination	536	42
Family income (*n* = 1,312)			Local anesthesia	186	15
Low	119	9	Extraction	448	35
Middle	933	71	Filling	565	44
High	260	20	Prophylaxis	189	15
Mother’s education (*n* = 1,308)			Other	82	6
Less than secondary	334	26	Behavior during previous dental visit/s (*n* = 1,295)^b^		
Secondary	508	39	Good	744	57
University	466	36	Poor	444	34
Father’s education (*n* = 1,289)			I don’t know	107	8
Less than secondary	200	16	Fear (CFSS-DS) (*n* = 1,312)		
Secondary	449	35	Fearful (score ≥32)	265	20
University	640	50	Non-fearful	1,047	80
General health variables			Untreated caries (*n* = 1,312)		
Health problems (*n* = 1,269)			Yes	1,012	77
Yes	188	15	No	300	23
No	1,081	85	DMFT (mean (SD))	4.0 (3.2)	
Hospitalization (*n* = 1,283)					
Yes	308	24			
No	975	76			

*Abbreviations*: *DMFT* Decayed Missing and Filled Permanent Teeth; *CFSS-DS* Children’s Fear Survey Schedule-Dental Subscale; *SD* Standard deviation.

^a^Answer categories are not mutually exclusive

^b^Reported by parents

Table 1 summarizes the patterns of dental visits, behavior and fear among the study population. Approximately 90% of the children had ever visited a dentist, and 33% had visited the dentist in the last 3 months. The main reason for the most recent dental visit was pain (61%), followed by fillings (36%). The main procedures undergone at the previous dental visits were fillings (44%), examinations (42%) and extractions (35%). Approximately 34% of the parents reported that their child behaved poorly during their previous visits, and according to the CFSS-DS, 20% of the children were fearful. Regarding caries, 77% of the children had untreated caries, and the mean DMFT was 4.0 ± 3.3.

### Bivariate analysis

Table 2 shows the association between total CPQ_11–14_scores and sociodemographic and health-related characteristics. The CPQ_11–14_ scores ranged from 0 to 100. The mean ± standard deviation of CPQ_11–14_ score was 24.6 ± 17.2 while the median and interquartile range (IQR) was 21 (12–34). Regarding sociodemographic characteristics, the CPQ_11–14_ scores were significantly associated with the number of siblings, family income, the mother’s education and the father’s education (*P*-value < 0.001). In addition, higher mean scores were significantly associated with the presence of health problems (*P*-value < 0.001) and prior hospitalization (*P*-value = 0.001). The CPQ_11–14_ scores were not significantly affected by age, gender or birth order.

**Table 2:** Total OHRQoL (CPQ_11–14_) scores by sociodemographic and general health variables

Sociodemographic variables	Mean	SD	Median	IQR	*P*-value^a^
Total score	24.6	17.2	21	12–34	---
Age (years)					
11-13	25.1	17.2	21	12–33	0.328
14-15	24.2	17.2	20	12–35	
Gender					
Male	25.0	17.6	21	12–35	0.357
Female	24.2	16.7	20	12–33	
Birth order					
First	24.5	17.0	20	12–34	0.156
2-3	23.7	17.1	20	11–33	
4+	25.7	17.3	23	12–36	
Number of siblings					
0-3	23.3	16.5	19	12–32	0.002^b^
4-5	23.8	17.0	20	11–32	
6+	27.2	18.0	24	13-37	
Family income					
Low	30.3	19.7	27	15–43	<0.001^c^
Middle	24.6	16.9	21	12–34	
High	22.2	16.6	19	10–31	
Mother’s education					
Less than secondary	27.0	18.4	22	13–37	0.007^d^
Secondary	24.3	16.7	21	12–34	
University	23.2	16.6	19	12–31	
Father’s education					
Less than secondary	29.3	18.8	28	14–41	<0.001^e^
Secondary	24.0	17.0	20	12–33	
University	23.7	16.5	20	12–33	
General health variables					
Health problems					
Yes	28.3	18.4	26	14–39	<0.001
No	23.7	16.6	20	12–33	
Hospitalization					
Yes	27.2	17.8	23	14–38	0.001
No	23.7	16.8	20	12–33	

*Abbreviations*: *OHRQoL* Oral health related quality of life; *CPQ*_*11–14*_ Child Perceptions Questionnaire for 11- to 14-year-olds; *SD* Standard deviation; *IQR* Interquartile range.

^a^Two-sample *t*-test, One-way ANOVA test and Tukey post-hoc test were used

^b^0–3 < 6+; 4–5 < 6+

^c^High < Low; Middle < Low

^d^University < Less than secondary

^e^Secondary < Less than secondary; University < Less than secondary

Table 3 presents the associations between total CPQ_11–14_ scores and the patterns of dental visits, behavior and fear. The children who reported pain as the main reason for their most recent dental visit reported significantly higher CPQ_11–14_ scores than those who did not (*P*-value < 0.01). In addition, the children who reported that the main procedure at their previous dental treatment was an extraction or filling had higher scores than those who did not (*P*-value < 0.05). The children whose parents reported that the child behaved poorly at previous dental visit/s had higher scores than those whose parents reported good behavior (*P*-value < 0.001). Fearful children reported higher CPQ_11–14_ scores compared with non-fearful ones (*P*-value < 0.001). CPQ_11–14_ scores had a weak, positive and non-significant correlation with DMFT scores (*ρ* = 0.03, *P*-value = 0.356) and were not significantly associated with untreated caries.

**Table 3:** Total OHRQoL (CPQ_11–14_) scores by patterns of dental visits, behavior and fear among the study population

Dental history & experience	Mean	SD	Median	IQR	*P*-value^a^
Dental visit ever					
Yes	24.7	17.2	21	12–34	0.855
No	25.0	17.8	20	12–35	
Dental visit in the last 3 months					
Yes	24.3	16.9	21	12–34	0.674
No	24.7	17.3	20	11–34	
Reason for most recent dental visit					
Regular check-up					
Yes	23.0	16.9	18	10–33	0.307
No	24.8	17.2	21	12–34	
Pain					
Yes	25.7	17.7	22	13–35	0.004
No	22.9	16.2	20	11–33	
Filling					
Yes	25.4	18.0	22	12–36	0.226
No	24.2	16.8	20	12–33	
Esthetic					
Yes	24.1	16.5	20	11–35	0.673
No	24.7	17.3	21	12–34	
Procedure at previous dental visit/s					
Examination					
Yes	24.3	16.2	21	12–34	0.549
No	24.9	17.9	21	12–35	
Local anesthesia					
Yes	25.9	18.2	22	12–36	0.279
No	24.4	17.0	21	12–34	
Extraction					
Yes	26.1	18.3	22	13–36	0.027
No	23.9	16.6	20	12–33	
Filling					
Yes	25.9	18.3	22	12–37	0.016
No	23.6	16.3	20	12–32	
Prophylaxis					
Yes	23.1	16.4	20	11–33	0.181
No	24.9	17.3	21	12–35	
Behavior during previous dental visit/s^b^					
Good	23.5	16.9	20	11–33	<0.001
Poor	26.9	17.9	23	14–37	
Fear (CFSS-DS)					
Fearful (score ≥32)	32.6	18.8	31	18–40	<0.001
Non-fearful	22.6	16.2	19	11–30	
Untreated caries					
Yes	24.8	17.3	21	12–34	0.648
No	24.2	16.8	20	12–35	
DMFT (Pearson’s ρ)	0.03				0.356

*Abbreviations*: *OHRQoL* Oral health related quality of life; *CPQ*_*11–14*_ Child Perceptions Questionnaire for 11- to 14-year-olds; *SD* Standard deviation; *IQR* Interquartile range; *CFSS-DS* Children’s Fear Survey Schedule-Dental Subscale.

^a^Two-sample *t*-test was used

^b^Reported by parents

### Regression analysis

The significant predictors of OHRQoL determined with linear regression are displayed in Table 4. Number of siblings, family income, parental education, health problems, hospitalization, dental fear, pain as the reason for the most recent dental visit, and filling as the procedure performed during the previous dental visits were significantly associated with OHRQoL. For every unit increase in the number of siblings, there was a 0.5-unit increase in the CPQ_11–14_ score (*P*-value < 0.05). Compared with children with a low family income, children with a high family income had CPQ_11–14_ scores that were 5.2 units lower (*P*-value < 0.01). Compared with children whose fathers had less than a secondary education, children whose fathers had a secondary or university education had CPQ_11–14_ scores that were approximately 4 units lower (*P*-value < 0.01). Regarding general health, those who reported a history of hospitalization or health problems had CPQ_11–14_ scores 3.1 units higher than those who did not (*P*-value < 0.05). Regarding dental history, those who reported pain as the main reason for their most recent dental visit had scores 2.2 units higher than those who did not (*P*-value < 0.05), and those who reported receiving a filling during previous dental visits had scores 2.7 units lower than those who did not (*P*-value < 0.01). Fear was a strong predictor of CPQ_11–14_ scores, and fearful children had scores 9.9 units higher than non-fearful children (*P*-value < 0.001).

**Table 4:** Linear regression results (coefficients, SEs, 95% CIs and *P*-values) for predictors of OHRQoL (CPQ_11–14_) scores

Significant Predictors	Coefficient	SE	95% CI	*P*-value
Number of siblings	0.5	0.2	(0.1, 0.9)	0.017
Income				
Low	Ref	---	---	---
Middle	−3.2	1.8	(−6.6, 0.3)	0.075
High	−5.2	2.0	(−9.1, −1.3)	0.009
Father’s education				
Less than secondary	Ref	---	---	---
Secondary	−4.3	1.5	(−7.1, −1.4)	0.004
University	−3.9	1.4	(−6.7, −1.0)	0.008
Ever hospitalized	3.1	1.1	(0.9, 5.3)	0.007
Health problem	3.1	1.1	(0.5, 5.7)	0.022
Dental fear (Fearful = 1)	9.9	1.2	(7.6, 12.2)	<0.001
Pain as reason for most recent dental visit	2.2	1.0	(0.3, 4.1)	0.011
Received a filling at previous dental visit/s	−2.7	0.9	(−4.5, −0.8)	0.005

*Abbreviations*: *OHRQoL* Oral health related quality of life; *CPQ*_*11–14*_ Child Perceptions Questionnaire for 11- to 14-year-olds; *SE* Standard error; *CI* Confidence interval

## Discussion

This school-based cross-sectional study investigated the effects of past dental history, dental experience, dental fear and sociodemographic factors on the OHRQoL of children aged 11 to 14 years. Our findings revealed that dental fear, pain as the reason for the most recent dental visit, a larger number of siblings, lower family income, a lower paternal education level, health problems and prior hospitalization were significantly associated with lower OHRQoL. Having received a filling during the previous dental visits was significantly associated with better OHRQoL.

In our study, dental fear was a strong and significant predictor of poor OHRQoL. Similarly, dental fear in 11- to 14-year-old children has been reported to negatively influence their OHRQoL [12]. This association could be explained by known risk factors that are associated with dental fear, such as irregular dental visits and high numbers of caries. A previous survey conducted among Swedish adults found that dental anxiety was associated with irregular dental care and low OHRQoL [23]. In addition, fearful children (aged 4–11 years old) and fearful preschoolers were reported to have had more caries experience and more avoidance patterns than those without fear [24, 25]. Therefore, the early recognition and management of childhood dental fear is essential for delivering effective child dental treatment, reducing dental avoidance and behavior management problems and improving children’s overall oral health and OHRQoL. However, it must be noted that the relationship between dental fear and OHRQoL is likely to be bi-directional as poor OHRQoL could also cause or exacerbate dental-related fear.

Children who reported pain as the main reason for their most recent dental visit reported significantly higher CPQ scores than those who did not. In a previous study, two-thirds of 11- to 14-year-olds visited the dentist because of pain that had a significant impact on their daily activities [26]. Dental pain has been reported to be significantly associated with poor OHRQoL in 10- to 19-year-olds [27] and 8- to 12-year-olds [28]. Dental pain is highly likely to be an indicator of dental caries [26–28], and sporadic dental treatment prompted by pain usually requires invasive procedures and extractions that are associated with fear [29]. School-based preventive strategies should be encouraged to reduce the prevalence of caries and dental pain and to improve OHRQoL.

Receiving a dental filling during the previous visits was a significant predictor of better OHRQoL. This finding is in line with previous studies that found significant improvement in OHRQoL in 12-year-old children who had undergone dental treatment compared with children who had untreated carious teeth [30] and in preschoolers after complete dental treatment [31]. Such results are expected, as restoration of teeth eliminates pain and restores function.

The pattern of dental visits did not show any significant effect on the OHRQoL. This finding is surprising, particularly because irregular dental visits have been reported to be associated with more numerous caries and untreated caries [32, 33]. Our findings also contradict other studies, in which poor OHRQoL was associated with not having visited the dentist in the previous year [10]. Afonso-Souza et al. [9] suggested that subjects with a high socioeconomic status who have good oral health usually make regular dental visits, unlike subjects with a low socioeconomic status and poor oral health. They added that any protective effect of regular visits on perceived oral health that they found in their results would be small or null because of residual confounders. The effect of the pattern of previous dental visits remains unclear and needs further investigation.

We found several sociodemographic characteristics that were associated with poor OHRQoL; higher mean CPQ_11–14_ scores were significantly associated with a greater number of siblings, lower family income and parents with lower education. A recent longitudinal study found that the socioeconomic factors parental education and income were a consistent predictor of OHRQoL for 10- to 14-year-old children [34]. In addition, a Brazilian study reported that low family income, mothers with less than 9 years of education, and living with two or more siblings were significantly associated with poor OHRQoL [14]. Children of mothers with low education who did not visit the dentist regularly were found to be at risk of not receiving dental care [35]. In the present study, children who had fathers with low education had poor OHRQoL. This finding can be attributed to the fact that in the community in which the participants lived, the father is the earner in the family. In addition, taking children for dental care is the responsibility of the parents, and because oral health knowledge and beliefs are gained through education and reflected in attitudes and behaviors, fathers with low education might not value the need to seek early dental care for prevention and treatment.

Higher mean CPQ_11–14_ scores were significantly associated with previous health problems and a history of hospitalization. Poor self-reported oral health has been associated with poor general health in 12- and 15-year-olds [10]. A systematic review suggested that the deterioration of oral health in hospitalized patients could have an impact on their health and quality of life by increasing the risk of acquired infections [36]. The interaction between general health and oral health is bidirectional and complex. There is a potential for oral pathogens and their toxins to spread to other organs and tissues, while the drugs and techniques used to treat systemic diseases can produce a low salivary flow or xerostomia, which affects mastication, swallowing, taste, speech, halitosis and esthetics [37].

The CPQ_11–14_ scores had a weak, positive and non-significant association with DMFT scores and were not significantly associated with untreated caries. This finding contradicts a study that reported a significant association between caries and CPQ_11–14_ scores in 12-year-old Ugandan children [38]. A higher number of caries was significantly associated with higher CPQ scores for 8- to 10-year-old Australian children, but not 11- to 13-year-olds [13]. Similar to our findings, a UK study reported no association between DMFT in 11- to 14-year-olds and CPQ scores [39]. In addition, no association was found between caries and oral symptoms and the functional limitation domains of the CPQ_11–14_ in Brazilian 12-year-olds [14]. The authors of the Brazilian study attributed their findings to the low caries prevalence because approximately 60% of their sample had zero DMFT. However, in our study, a high percentage of the children (77%) had untreated caries. Our non-significant findings may be explained by the fact that 11- to 14-year-old children usually have newly erupted permanent teeth in which caries extension is limited and superficial and might not cause severe discomfort or functional impairments.

This study has some limitations. The OHRQoL is a complex construct and has a bidirectional relationship with many factors; consequently, the cross-sectional design limits the ability to draw causal inferences because the temporality of the reported relationships cannot be established. Self-reported questionnaires rely on the participants’ ability to understand the questions and the answer options provided. To overcome this limitation in a relatively young population, the questionnaire was provided to students who had at least 6 years of formal education in their native language, and a trained investigator was available to clarify any ambiguous item or answer any questions. This study only included 11- to 14-year-old children and excluded other age groups because the CPQ_11–14_ is the only scale that measures OHRQoL that has been validated in the Arabic language. Research is ongoing to validate scales used for other age groups, which will facilitate researchers’ investigations of OHRQoL in Arabic populations. Finally, although the study was conducted among a multi-stage stratified sample of school children living in Jeddah, Saudi Arabia, the results might not be generalizable to children living in other regions or other parts of the world.

## Conclusions

The present study confirmed the association between dental fear and previous dental experience and OHRQoL. Significant associations were observed between dental fear, pain as the reason for the most recent dental visit, a larger number of siblings, lower family income, lower paternal education levels, health problems and prior hospitalization and poor OHRQoL, while having received a filling during the previous dental visits was significantly associated with better OHRQoL. Patterns of previous dental visits and caries were not associated with OHRQoL. Nonetheless, fear plays an important role in dental practice, and the early recognition of fearful children is necessary to guide their behavior and overcome the effects of fear on their OHRQoL. In addition, oral health education should be directed toward low socioeconomic families to improve their children’s oral health and OHRQoL. Further studies are needed to assess the association between these factors and specific domains of OHRQoL. Longitudinal studies are also needed to study the cause and effect of different variables on OHRQoL.

## Additional file

Additional file: Parent Questionnaire: was directed to the children’s parents and used to assess the different factors associated with OHRQoL. (PDF 121 kb)

## Abbreviations

ANOVA: Analysis of variance
CFSS-DS: Children’s Fear Survey Schedule-Dental Subscale
CPI: Community Periodontal Index
CPQ: Child Perceptions Questionnaire
DMFT: Index for decayed, missing and filled permanent teeth
Dmft: Index for decayed, missing and filled primary teeth
OHRQOL: Oral health related quality of life
