# Supplementary material for: Do children’s previous dental experience and fear affect their perceived oral health-related quality of life (OHRQoL)?
Source: BMC Oral Health. 2017 Jan 16;17:47. doi: 10.1186/s12903-017-0338-9 (PMC5240375; doi:10.1186/s12903-017-0338-9)
Supplement: Additional file: — Parent Questionnaire: was directed to the children’s parents and used to assess the different factors associated with OHRQoL. (PDF 121 kb) [file 12903_2017_338_MOESM1_ESM.pdf]

King Abdulaziz University Faculty of Dentistry
Department of Pediatric Dentistry

Parents' Questionnaire

Name: ______________________

Age: _________ years

Gender: _______________

Grade: ________________

Please fill the following information about your child:

Number of sibling(s): ________________

Birth order of your child: ______________

The first questions will ask you information about your child:

1. Does your child have any chronic health conditions or is currently taking any medications?

a) Yes. *Please specify* ___________________________
b) No
c) I don't know

2. Has your child ever been hospitalized?

a) Yes. *Please specify* ___________________________
b) No
c) I don't know

2. How often does your child visit the dentist?

a) Regularly, every 6 months
b) When feeling pain only
c) Occasionally
d) He/she never visited a dentist
e) I don't know

4. In the last year, has your child visited the dentist?

a) Yes
b) No
c) I don't know

5. In the past 3 months, has your child visited the dentist?

a) Yes
b) No
c) I don't know

6. What were the reasons for his/her most recent dental visit? (check all that apply):

a) Regular checkup
b) Dental pain
c) Dental filling(s)
d) Esthetic reason(s)
e) Other(s)……………………………………..

7. What procedures did his/her previous dental treatment include? (check all that apply):

a) Dental examination
b) Local anesthesia
c) Dental extraction
d) Tooth filling
e) Dental prophylaxis
f) Other(s) ___________________________

8. During the previous dental visit(s), did he/she feel pain?

a) Yes
b) No
c) Slightly
d) I don't know

9. During the previous dental visit(s), how was his/her behavior?

a) Crying
b) Screaming
c) Resistant
d) Cooperative
e) Happy
f) I don't know

These next questions will ask you for information about the child's mother and father:

Question	Father	Mother
**What is your age?**		
**What is your nationality?**		
**What is your Current marital status?**	A) Married<br>B) Divorced/Separated<br>C) Widowed	A) Married<br>B) Divorced/Separated<br>C) Widowed
**What is the highest level of school you attended?**	A) Less than secondary<br>B) Secondary<br>C) University	A) Less than secondary<br>B) Secondary<br>C) University
**What is your current occupation?**	A) Laborer<br>B) Self-employed<br>C) Employee (Government or private)<br>D) Soldier<br>E) Unemployed<br>F) Student<br>G) Retired	A) Laborer<br>B) Self-employed<br>C) Employee (Government or private)<br>D) Unemployed<br>E) Student<br>F) Retired
**What is your current monthly family income?**	A) Less than 5,000 Saudi Riyals<br>B) Between 5,000 and 10,000 Saudi Riyals<br>C) More than 10,000 Saudi Riyals	

This questionnaire was filled by the child's:

A. Father
B. Mother
C. Both parents
D. Grandparent
E. Other (how is he/she related to the child?) ____________________________

Thank you for taking the time to complete this survey!
